# Training response inhibition to reduce food consumption: Mechanisms, stimulus specificity and appropriate training protocols

**DOI:** 10.1016/j.appet.2016.11.014

**Published:** 2017-02-01

**Authors:** Rachel C. Adams, Natalia S. Lawrence, Frederick Verbruggen, Christopher D. Chambers

**Affiliations:** a School of Psychology and Cardiff University Brain Research Imaging Centre, Cardiff University, Park Place, Cardiff CF10 3AT, UK; b School of Psychology, College of Life and Environmental Sciences, University of Exeter, Exeter EX4 4QG, UK

**Keywords:** Response inhibition, Cognitive training, Food intake, Dietary restraint, Implicit attitudes

## Abstract

Training individuals to inhibit their responses towards unhealthy foods has been shown to reduce food intake relative to a control group. Here we aimed to further explore these effects by investigating the role of stimulus devaluation, training protocol, and choice of control group. Restrained eaters received either inhibition or control training using a modified version of either the stop-signal or go/no-go task. Following training we measured implicit attitudes towards food (Study 1) and food consumption (Studies 1 and 2). In Study 1 we used a modified stop-signal training task with increased demands on top-down control (using a tracking procedure and feedback to maintain competition between the stop and go processes). With this task, we found no evidence for an effect of training on implicit attitudes or food consumption, with Bayesian inferential analyses revealing substantial evidence for the null hypothesis. In Study 2 we removed the feedback in the stop-signal training to increase the rate of successful inhibition and revealed a significant effect of both stop-signal and go/no-go training on food intake (compared to double-response and go training, respectively) with a greater difference in consumption in the go/no-go task, compared with the stop-signal task. However, results from an additional passive control group suggest that training effects could be partly caused by increased consumption in the go control group whereas evidence for reduced consumption in the inhibition groups was inconclusive. Our findings therefore support evidence that inhibition training tasks with higher rates of inhibition accuracy are more effective, but prompt caution for interpreting the efficacy of laboratory-based inhibition training as an intervention for behaviour change.

## 1 Introduction

Obesity rates have risen sharply over the last few decades, creating a global epidemic with gross implications for personal and economic health (e.g. Bray, 2004, Fry and Finley, 2005, Mokdad et al., 2003). One of the common explanations for the obesity epidemic is the environment and the availability of highly varied, palatable and fattening foods (Cummins and Macintyre, 2006, French et al., 2001, Jeffery and Utter, 2003, Levitsky, 2005, McCrory et al., 1999). However, despite the ‘obesogenic environment’ in which we live, there is considerable variation in weight status across individuals. For some, the ability to resist such tempting foods remains a constant challenge, whereas others find it relatively easy to exercise self-control over their calorie intake and maintain a healthy lifestyle. This leads us to the question of why some individuals are able to succeed where others fail?

Dual process models argue that our behaviour is determined by the interaction of an impulsive system, which is driven by our hedonic needs, and a reflective system, which involves conscious thought and deliberation (Strack & Deutsch, 2004). In the case of overeating it is possible that vulnerable individuals possess strong impulsive desires for calorie-dense foods and a lack of control over these desires (e.g. Houben et al., 2012b, Lawrence et al., 2012, Price et al., 2016, Volkow et al., 2003, Volkow et al., 2002,; White, Whisenhunt, Williamson, Greenway, & Netemeyer, 2002; for a review see; Stice, Lawrence, Kemps, & Veling, 2016). This has encouraged researchers to develop new behavioural interventions designed to target these processes. For example, recent studies have shown that training individuals to inhibit simple motor responses to images of food, using either the stop-signal or go/no-go tasks, can result in the decreased consumption of that food (Houben and Jansen, 2011, Houben and Jansen, 2015, Houben, 2011, Lawrence et al., 2015a, Veling et al., 2011), healthier food choices (Koningsbruggen et al., 2014, Veling et al., 2013a, Veling et al., 2013b) and even weight loss (Lawrence et al., 2015b, Veling et al., 2014). Three recent meta-analyses have demonstrated small to medium effect sizes for the effect of food-related inhibition training compared to control training (Allom et al., 2015, Jones et al., 2016; Turton, Bruidegom, Cardi, Hirsch, & Treasure, 2016). However, there are some inconsistencies in training effects and several questions remain unanswered. In the two studies presented here, we sought to investigate the mechanisms involved in these training effects and whether such effects are reliant upon stimulus-specific associations between the stop-signal and the trained food. For example, inhibition training may be most effective when strong automatic associations are formed between the foods and a successful stop response (Jones et al., 2016). Stimulus-specific training effects would therefore result in reduced consumption of the trained foods only, whereas generalised effects could see reduced consumption of other unhealthy foods or even healthy foods. Following from the results of Study 1, we also compared the effectiveness of two different training protocols, stop-signal and go/no-go, and investigated the consumption of both unhealthy and healthy foods following training. Furthermore, we examined whether these training effects were the result of reduced food consumption in the training groups or whether they could be attributed to increased consumption in the ‘food-go’ control groups.

## 2 Study 1

In Study 1 we investigated whether stop-signal training could reduce food consumption relative to a control task, and whether any effects of training were due to the devaluation of inhibited stimuli (i.e. a reduction in the perceived incentive value or attractiveness of the stimulus). It has been argued that the inhibition of responses towards a desired object can result in stimulus devaluation (Doallo et al., 2012, Frischen et al., 2012, Verbruggen et al., 2014a, Wessel et al., 2014), a process that may occur in order to resolve action conflict (Veling, Holland, & van Knippenberg, 2008) or due to the inherent links between avoidance and aversion (McLaren and Verbruggen, 2016, Verbruggen et al., 2014b). Lawrence et al. (2015b) and Veling et al. (2013b) have both provided evidence for inhibition-induced stimulus devaluation when they found that foods paired with inhibition during a food-related go/no-go task were rated less positively than foods associated with responding. Changes in *implicit* attitudes have also been shown to mediate the effect of alcohol-related go/no-go training on weekly alcohol intake in heavy drinkers (Houben et al., 2012a, Houben et al., 2011). To date, evidence for the stimulus devaluation hypothesis is equivocal (e.g. Bowley et al., 2013, Houben et al., 2012a), with a recent meta-analysis showing no evidence for an effect of inhibition training on stimulus devaluation across six food and alcohol-related inhibition training studies (Jones et al., 2016). However, there are several gaps in this literature, with no published studies exploring the effect of alcohol-related inhibition training on explicit attitudes or food-related inhibition training on implicit attitudes. In addition, there are no studies investigating whether food or alcohol stimuli are devalued following stop-signal (as opposed to go/no-go) training.

The aim of Study 1 was to address one of these gaps by training participants on a food-related stop-signal task and measuring both implicit attitudes towards food and food consumption. In accordance with previous suggestions that inhibition training is most effective for those with a strong impulsive desire towards food (Veling et al., 2011), we restricted our sample *a priori* to participants who scored highly on measures of chocolate craving and dietary restraint. This sample has also previously been shown to respond positively to go/no-go training (Houben & Jansen, 2011). Participants were randomly allocated to either a stop training or control group. Those in the stop group performed a stop-signal task in which they had to inhibit their responses to chocolate stimuli on the majority of trials, whereas those in the control group made an additional response on chocolate trials (double-response group). As the presentation of the stop signal in the stop-signal task requires not only response inhibition but also additional error monitoring, rule maintenance, attentional control and response selection processes, this double-response task was believed to be an appropriate control condition (Lawrence et al., 2015a, Tabu et al., 2011, Verbruggen et al., 2012, Verbruggen et al., 2010, Wessel et al., 2014).

Compared to the double-response group, it was hypothesised that participants in the stop group would show a reduced positive implicit attitude and/or an increased negative implicit attitude towards chocolate (measured using two unipolar, Single-Category Implicit Association Tests [SC-IAT]; Greenwald et al., 1998, Houben et al., 2010, Karpinsky and Steinman, 2006 and also that they would consume fewer calories in a bogus taste test. Stimulus-specific effects on food intake were also investigated by including crisps in both the training task as ‘go’ foods (presented alongside stop-signals on a minority of trials) and the taste test. In accordance with previous research we expected that the stop group would show reduced consumption of the chocolate only (Houben, 2011; Lawrence et al., Study 2, 2015a; Veling et al., 2013a). Any effect of training on crisp intake could imply the occurrence of underlying mechanisms other than stimulus devaluation such as an increase in general self-control or response inhibition (Baumeister et al., 2006, Berkman et al., 2009, Berkman et al., 2012, Jones et al., 2011, Muraven, 2010, Verbruggen et al., 2012).

### 2.1 Method

**Fig. 1:**
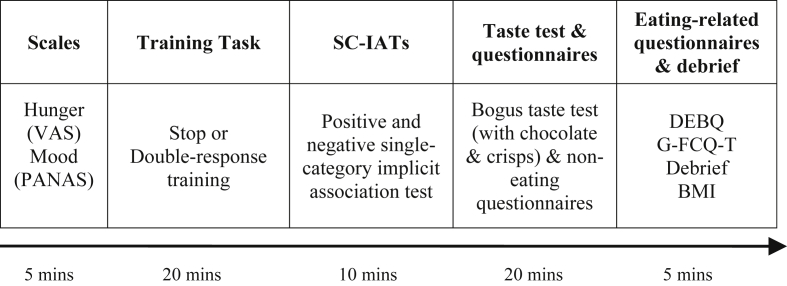
Schematic diagram of the procedure for Study 1. Participants completed measures of hunger and mood states before starting the training task. Participants were randomly allocated to either the stop (inhibition) or double-response (control) training. Following this task participants were presented with two SC-IATs to measure positive and negative implicit attitudes towards chocolate (in a counterbalanced order). After the computer tasks participants were presented with a taste test and a battery of personality questionnaires. After 20 min had elapsed the food was removed and participants completed further eating-related questionnaires before being debriefed (all details can be found in the Method section).

Fig. 1 provides a schematic diagram of the procedure for Study 1.

#### 2.1.1 Participants

One hundred and forty-three restrained (15 + on the Restraint Scale; Herman & Polivy, 1980) chocolate cravers (10 + on the Attitudes to Chocolate Questionnaire Craving Scale; Benton, Greenfield, & Morgan, 1998, p. 134 females; aged 18–61, *M* = 22.92, *SE* = 0.68) were pseudo-randomly divided into the stop (*n* = 71, 66 females) and double-response groups (*n* = 72, 68 females) with an attempt to keep age and gender evenly distributed. Sample size was determined according to a Bayesian inferential stopping rule for the main effect of total calorie intake between groups; see Statistical Analysis in the Supplementary Information. Participants were recruited from the staff and student population at Cardiff University and were not eligible if they were currently dieting (with a weight goal and timeframe in mind) or if they had any history of eating disorders. All participants, in both studies 1 and 2, were reimbursed for their participation; they received course credit or were offered either £6 or entry into a prize draw (for a £100 Amazon voucher). Both studies were approved by the Research Ethics Committee at the School of Psychology, Cardiff University.

#### 2.1.2 Materials/measures

##### 2.1.2.1 Training task

Stimuli consisted of eight images of chocolate, eight images of crisps and 32 filler images (household items). All images were close up shots of the item presented against a white background. All images and experimental materials are available from the authors on request.

**Fig. 2:**
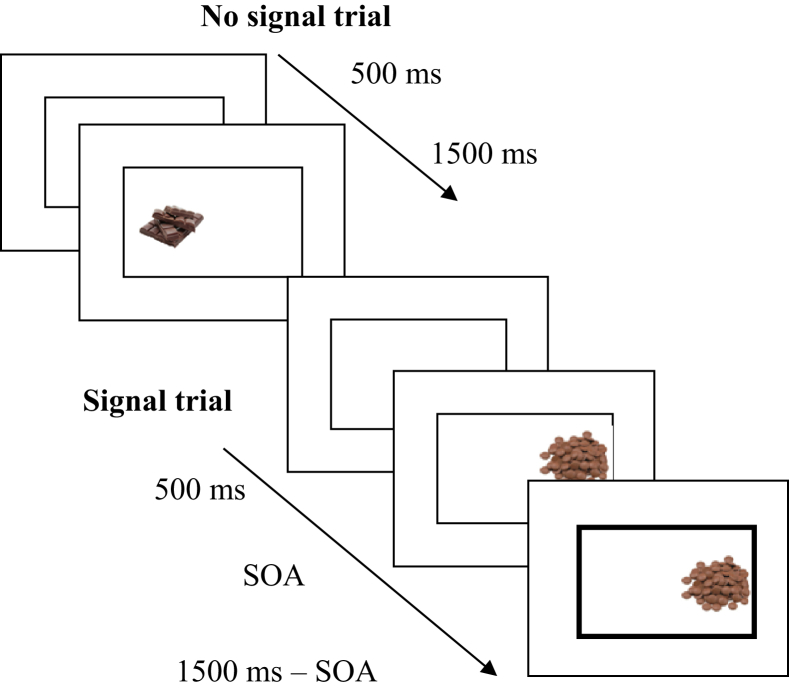
Display sequence for the training task. For no-signal trials participants were presented with a rectangle for fixation before seeing a stimulus appear on the left or right hand side. On no-signal trials (i.e. when the lines of the rectangle remained thin), participants were instructed to respond to the stimulus location using the ‘C’ and ‘M’ keys, with their left and right index fingers, respectively. For signal trials, the lines of the rectangle turned bold after a variable delay (SOA), which was initially 250 ms and was then adjusted using a simulated tracking procedure (see Training Task). On signal trials, participants were asked to either inhibit their response (stop group) or make an additional response (double-response group).

In each trial a rectangle appeared in the centre of the screen (fixation). After 500 m, a stimulus appeared in the left or right side of the rectangle and remained on screen for 1500 m (see Fig. 2). Participants were asked to respond to the location of the stimulus (no-signal trial) as quickly and accurately as possible, using their left and right index fingers (‘C’ or ‘M’, respectively, on a standard QWERTY keyboard). On a subset of trials (120 of 480 trials; 25%), the rectangle would turn bold after a variable delay (stimulus-onset asynchrony; SOA). The instructions for signal trials depended on the training condition. Participants in the stop group were instructed to withhold their responses when a signal occurred, whereas those in the double-response group were instructed to make an additional response by pressing the space bar with their thumb when a signal occurred. The SOA was initially set at 250 ms and was then continuously adjusted using a simulated tracking procedure (see Lawrence et al. (2015a) for details). Signals were presented on the majority of chocolate trials (70 of 80 in total; 87.5%) to encourage learning of chocolate-stop associations in the stop group. Images of crisps (10 of 80; 12.5%) and filler images (40 of 320; 12.5%) were also presented alongside a signal on a minority of ‘catch’ trials.

The task consisted of ten blocks of 48 trials and lasted approximately 20 min. Each image was presented once per block and the stimulus type and location of the image were randomly intermixed with equal probability. At the end of every block participants were given a 10 s break and were provided with feedback. Those in the stop group were asked to speed up or slow down their responses depending on their stop performance. If the percentage of failed inhibition trials exceeded 70% they were asked to “SLOW DOWN” and if it was less than 30% they were asked to “SPEED UP”. If performance was between 60 and 70% or between 30 and 40% they were asked to respond “a little slower” or “a little faster”, respectively. When performance was between 40 and 60% they saw the message “Good!!”. Participants in the double-response condition were only informed if they had missed more than three double-responses, otherwise they were shown the message “Good!!”. If participants missed more than three no-signal responses they were also provided with feedback to respond on all no-signal trials. This feedback, along with the simulated tracking procedure and catch trials, were included to maintain task difficulty and to ensure that the task remained a stop-signal task (with demands on ‘action cancellation’), rather than becoming a go/no-go task (which mostly requires ‘action restraint’; Eagle et al., 2008, Schachar et al., 2007). The researcher was present throughout the training phase and observed the first block to ensure that participants understood the task instructions and were responding correctly on signal trials.

All computerised tasks (for both Study 1 and Study 2) were programmed in Matlab (Mathworks, Natick, MA) using Psychophysics Toolbox (www.psychtoolbox.org) and were presented on a 19-inch flat-panel LCD monitor.

##### 2.1.2.2 Unipolar, single-category implicit association test (SC-IAT)

All participants performed two unipolar SC-IATs (Greenwald et al., 1998, Houben et al., 2010, Karpinsky and Steinman, 2006): a unipolar positive SC-IAT and a unipolar negative SC-IAT. For both tasks the target category was chocolate (six images of chocolate; label ‘chocolate’). Three of the chocolate images were the same as those used in the training procedure and the remainder had not been seen before. For the positive SC-IAT the attribute categories were pleasant (words: delicious, delightful, great, heavenly, outstanding, tasty; label ‘pleasant’) and neutral (words: adequate, average, general, moderate, ordinary, undefined; label ‘neutral’), for the negative SC-IAT the attribute categories were unpleasant (words: awful, bad, disgusting, horrible, nasty, revolting; label ‘unpleasant’) and neutral.^1^

Both tasks consisted of three blocks. The first block was for participants to practice categorising the attribute categories (24 trials). Participants were instructed to categorise the words as quickly and as accurately as possible using their left and right index fingers (‘C’ and ‘M’ response keys on a standard keyboard). In the second block, chocolate stimuli were paired with one of the attribute categories and were categorised using the same response keys (e.g. chocolate + pleasant vs neutral in the positive SC-IAT). The response assignment of the target category was then reversed in the third block (e.g. pleasant vs neutral + chocolate). There were 72 trials in both the second and third blocks. A 5:2:5 ratio was used to keep the number of responses on each key comparable, so that chocolate images were repeated five times (30 trials), attributes paired with chocolate were repeated twice (12 trials) and attributes not paired with chocolate were repeated five times (30 trials; Houben et al., 2010). Each block was preceded by a set of instructions regarding the appropriate responses. Attribute labels were presented throughout the blocks to the bottom-left and bottom-right of the screen and all stimuli appeared in the centre of the screen. All stimuli remained on screen until a response was given, or for 1500 m. Participants were provided with feedback after every trial: a green or red circle appeared for 150 m in the centre of the screen for correct and incorrect responses, respectively; if they failed to respond in time the message ‘too slow!’ appeared for 500 ms.

The order of the SC-IATs was counterbalanced across participants (positive-negative or negative-positive). The assignment of the target category was also counterbalanced so that half the sample received the target category pairing block (pleasant or unpleasant + chocolate *vs.* neutral) followed by the neutral category pairing block (neutral + chocolate *vs.* pleasant or unpleasant), whereas the other half received them in the reverse order. The assignment of the attribute categories to response keys was also counterbalanced.

##### 2.1.2.3 Taste test

Food consumption was measured using a bogus taste test. Participants were presented with two bowls containing milk chocolate buttons (∼210 g; *M* = 212, *SE* = 0.25; Tesco milk chocolate buttons, 5.4 kcal/g) and ready salted crisps (∼100 g; *M* = 101, *SE* = 0.25; Tesco ready salted crisps, 5.5 kcal/g; as in Lawrence et al., 2015a) and were also offered a cup of water (∼150 g; *M* = 151.12, *SE* = 0.73). Participants were told that we were interested in how their taste perceptions influenced the data and were invited to consume as much as they liked as the food would be thrown away after the study. They were provided with a questionnaire containing open-ended questions related to the taste of the products and Likert scales measuring the palatability and frequency of consumption for the two foods (this questionnaire was identical to that used in previous studies; Lawrence et al., 2015a, Houben, 2011). Participants were then left alone with the foods for 20 min while they completed a battery of personality questionnaires.^2^ They were left alone in a lab room without windows, for the duration of the taste test to minimise social influences on food intake (e.g. Roth, Herman, Polivy, & Pliner, 2001). The food products were weighed before and after the taste test without the participants’ knowledge. The difference in weight was converted to calories by multiplying the weight consumed by the caloric density of the food.

#### 2.1.3 Procedure

In order to control for levels of appetite, participants were asked to eat something small 3 h prior to the study and then to refrain from consuming anything except water during this time. This approach was consistent with previous studies (Guerrieri et al., 2009, Lawrence et al., 2015a) and was used to increase and standardise food appetite and motivation (Gibson and Desmond, 1999, Veling et al., 2013a, 2013b). Testing therefore only took place between 12 and 7 pm; this timeframe also coincides with an increase in food cravings (Hill, Weaver, & Blundell, 1991). After providing consent, participants answered questions regarding their hunger and mood states. They completed three 100 mm visual analogue scales (Flint et al., 2000, Yeomans, 2000) to assess feelings of hunger, fullness and desire to eat. They then completed the Positive and Negative Affect Schedule (PANAS; Watson, Clark, & Tellegen, 1988) to measure their current mood. These measures were taken at this stage as hunger and mood have been shown to be reliable predictors of food intake (Nederkoorn et al., 2009, Tice et al., 2001); it was important therefore to ensure that there were no group differences prior to the taste test. Participants then completed the training task and unipolar SC-IATs before the taste test and questionnaires. At the end of the study participants were debriefed and their awareness of the study's aims and stimulus mappings was assessed using open-ended, funnelled questions (full details of the debrief questions and statistical analyses are presented in the Supplementary Information). Participants' height and weight was measured to calculate BMI (kg/m^2^).

### 2.2 Statistical analyses

All demographic, state and trait variables were analysed to ensure that there were no statistically significant differences between training groups. Data from the training task were analysed to ensure that participants were performing the task as expected. IAT effects were calculated based on the *D*-score algorithm (see Supplementary Information for details). Following exclusions there was a final sample of 139 participants: 70 in the stop group and 69 in the double-response group. One participant from the double-response group was also excluded from the unpleasant SC-IAT analyses because their error rate was higher than a pre-set error criterion of 20% (Karpinsky & Steinman, 2006). All statistical analyses details, including sensitivity analyses and Bayes factors are presented in the Supplementary Information. Analyses of group differences and training data, along with additional analyses of SC-IATs and food consumption are also presented in the Supplementary Information. All study data is available online.^3^

### 2.3 Results

#### 2.3.1 Group differences

The two training groups were well-matched for all demographic, state and trait measures (see Supplementary Information for details).

#### 2.3.2 Unipolar, SC-IAT data analysis

Results from the SC-IAT analyses revealed only a significant main effect of SC-IAT type, with a positive score on the positive/pleasant SC-IAT (*M* = 0.42; *SE* = 0.04) and a negative score on the negative/unpleasant SC-IAT (*M* = −0.04; *SE* = 0.04; *p* < 0.001, *ƞ*^2^_p_ = 0.33). With the findings from the one-way *t*-tests, these results indicate that participants demonstrated a significant positive attitude towards chocolate on the pleasant SC-IAT (i.e. the presence of an implicit association between pleasant words and images of chocolate) but did not show a significant attitude in either direction on the unpleasant SC-IAT (i.e. no implicitly held association between unpleasant words and images of chocolate). The interaction between training condition and SC-IAT was not significant (*p* = 0.55, *ƞ*^2^_p_ = 0.003). Full analyses for the SC-IAT are presented in the Supplementary Information.

#### 2.3.3 Consumption data analysis

A 2 × 2 mixed ANOVA [between-subjects factor: *training condition* (stop or double-response); within-subjects factor: *food type* (chocolate and crisps)] revealed a significant main effect of food type showing that participants ate significantly more calories from chocolate (*M* = 217.95, *SE* = 13.5) than crisps (*M* = 152.99, *SE* = 9.54; *p* < 0.001, *ƞ*^2^_p_ = 0.2). However, the main effect of training condition (*p* = 0.16, *ƞ*^2^_p_ = 0.01) and the interaction between training condition and food type (*p* = 0.25, *ƞ*^2^_p_ = 0.01) were both non-significant. Contrary to the primary hypothesis, the *total* calorie consumption in the stop group was greater than that for the double-response group. Due to the direction of results, a Bayesian analysis (mean difference = -66.13, *SE* of the difference = 39.09) revealed a Bayes factor of 0.28, indicating substantial evidence for H0, which stated that stop training would *not reduce* calorie intake (B < 0.33; Dienes, 2011, Dienes, 2014). For completeness, a JZS Bayes factor was also calculated: this returned a Bayes factor of 0.67, indicating weak evidence in favour of H0.^4^

### 2.4 Discussion

The results of Study 1 revealed no significant effects of training on either implicit attitudes or food consumption. Moreover, the results for total food consumption were in the opposite direction to that predicted, and a Bayesian inferential analysis demonstrated that the results support the null hypothesis, relative to the alternative hypothesis of a stopping-induced reduction in consumption (Dienes, 2011, Dienes, 2014). Similarly, results for implicit attitudes were largely in favour of the null hypothesis (Rouder, Speckman, Sun, Morey, & Iverson, 2009). These results are inconsistent with our predictions based on previous studies that have revealed significant effects of inhibitory control training on food consumption (Allom et al., 2015, Houben, 2011, Houben and Jansen, 2011, Houben and Jansen, 2015, Lawrence et al., 2015a, Lawrence et al., 2015b, Veling et al., 2011). They are also in disagreement with predictions based on other studies showing effects of alcohol-related go/no-go training on implicit attitudes towards alcohol (Houben et al., 2011, Houben et al., 2012a). However, these null results support two previous food stop-signal training studies that also found no training effects (Allom and Mullan, 2015, Forman et al., 2016). Why is it that, overall, there are effects of food inhibition training on food intake (Allom et al., 2015, Jones et al., 2016; Turton et al., 2016) yet some studies fail to observe these effects? This study, like the other ‘failed’ attempts share several methodological differences with previous research, which should provide insight into these conflicting findings and guide future research towards more effective training protocols.

Critically, the results of Jones et al.’s (2016) meta-analysis showed that the proportion of successful inhibitions for target food trials, but not the absolute number of food-inhibition trials nor the contingency between the stimulus and stop signals, was predictive of inhibition training effects. The training protocol used in the present study may have failed to induce a sufficiently high proportion of successful stop responses to chocolate. Our stop-signal training task had an overall stop-signal rate of 25%, and paired a target food, chocolate, with signals on 87.5% of trials. However, the inclusion of inter-block feedback to ensure that training placed demands on action cancellation, as opposed to action restraint (Eagle et al., 2008, Schachar et al., 2007; see above), is likely to have decreased the rate of successful inhibition (70%) compared with other training tasks where the rate of inhibition was much higher (average ∼92%; Houben, 2011, Houben and Jansen, 2011, Houben and Jansen, 2015, Lawrence et al., 2015a, Lawrence et al., 2015b, Veling et al., 2011). In a recent study Forman et al. (2016) also included feedback in their stop-signal training task and found no independent effect of training on snack consumption. It is possible, therefore, that feedback and lower food-stop contingencies contributed to a reduction in successful stopping and hindered associative links between chocolate and inhibition, or more generally between unhealthy foods and inhibition, from developing (Verbruggen and Logan, 2008a, Verbruggen and Logan, 2008b, Verbruggen et al., 2014b, Verbruggen et al., 2014a).

This difference in associative learning may also explain why results appear to be more replicable and robust across inhibition training studies involving the go/no-go task, compared to the stop-signal task (Allom et al., 2015, Jones et al., 2016). Unlike the stop-signal task, the go/no-go task presents the stimulus and the signal at the same time, and go/no-go training studies have paired no-go signals consistently with target foods (i.e. 100% mapping; Houben and Jansen, 2011, Houben and Jansen, 2015, Lawrence et al., 2015b; van Koningsbruggen et al., 2014, Veling et al., 2011, 2013a, 2013b, 2014). These differences are associated with higher probabilities of stopping than in the stop-signal task (∼98%; Jones et al., 2016) and are therefore likely to influence the strength of stimulus-stop response associations (Verbruggen and Logan, 2008a, Verbruggen and Logan, 2008b). It is also thought that training on the go/no-go task is more likely to result in automatic inhibition compared to training on the stop-signal task (Spierer et al., 2013, Verbruggen et al., 2014a, 2014b). The results of Study 1 are therefore consistent with the conclusions of Jones et al., and suggest that developing strong associations between food and a stop response is key to the success of inhibition training studies.

Another probable factor contributing to the lack of effect on food consumption here is the intermediate SC-IAT tasks; it is possible that both the time taken to complete the tasks (∼10 min) and the nature of the response format in the tasks weakened any training-induced improvements in behavioural control. Previous studies investigating the effect of inhibition training on food intake have typically presented participants with a taste test immediately after training (Houben and Jansen, 2011, Houben, 2011, Lawrence et al., 2015a), although some studies have shown effects of inhibition training on food intake over a longer duration (Veling et al., 2011) and despite similar intermediate tasks (Houben and Jansen, 2015, Veling et al., 2013b). For example, Veling et al. (2013b) found that an intermediate task measuring explicit attitudes towards food did not negate the effect of food-related go/no-go training on food choice, and even mediated this effect. Explicitly evaluating food stimuli, however, is qualitatively different to the SC-IAT presented here where participants are asked to repeatedly pair chocolate with either pleasant or unpleasant words. It could be argued therefore that the SC-IAT acted as a form of evaluative conditioning and weakened the effects of inhibition training. Future research should consider using other measures of implicit attitudes, such as evaluative priming (e.g. Fazio et al., 1986, Lamote et al., 2004), to avoid this potential issue with the IAT – although any task requiring rapid responses to food may act to hinder any effects of inhibition training.

In conclusion, the results of Study 1 show that inhibition training may not be an effective intervention for reducing food consumption under all training conditions, but rather suggests that the training protocol must facilitate learning of stimulus-stop associations. This can be achieved by increasing the likelihood of participants successfully inhibiting their responses on target trials. For the stop-signal task in the present study, this would require the removal of inter-block feedback, increasing the proportion of stimulus-stop trials or changing the staircase tracking procedure; however, three recent meta-analyses have also indicated that training using the go/no-go task may be more effective at improving health behaviours compared to stop-signal training (Allom et al., 2015, Jones et al., 2016; Turton et al., 2016). The aim of Study 2, therefore, was to directly compare these training tasks in order to establish the most effective training protocol.

## 3 Study 2

The primary aim of Study 2 was to provide the first direct comparison between the stop-signal and go/no-go training protocols within one study. In addition, there were two further aims; the second aim was to explore the possibility that the training effects observed in previous studies are due to increased consumption in the ‘go’ control groups rather than a decrease in consumption in the inhibition groups; and finally we wanted to investigate the consumption of both healthy and unhealthy foods following training. Exploring the effect of training on healthy food consumption is not only of interest in terms of stimulus-specificity, but previous results have also suggested that training could be beneficial for improving healthy food behaviours (Blackburne et al., 2016, Veling et al., 2013a). An effect of training on both reduced unhealthy food consumption and increased healthy food consumption could be even more useful for any potential clinical applications of response inhibition training.

Although studies have focused on the use of inhibition training as a potential tool to reduce food consumption and aid weight loss (Lawrence et al., 2015a, Lawrence et al., 2015b, Veling et al., 2011, Veling et al., 2014), an alternative explanation for some of the observed effects is that they may in fact be due to increased consumption in the control groups rather than decreased consumption in the training groups. Previous studies investigating the effect of inhibitory control training on food intake have tended to include control conditions in which participants have been required to consistently respond to images of food (Houben, 2011, Houben and Jansen, 2011, Houben and Jansen, 2015, Lawrence et al., 2015a, Veling et al., 2011). It is possible that these ‘control’ conditions may act to increase dietary disinhibition as participants learn an approach response towards food. Schonberg et al. (2014) have also provided evidence for ‘go training’ effects; they showed that training participants to make a speeded cued response to unhealthy foods biased choice behaviour in favour of those foods. To test the direction of training effects in Study 2 we included an additional control group who simply observed the task; participants therefore received the same level of exposure to palatable foods but did not make or inhibit any responses (observe group). If both inhibition and approach processes occur in active versus control groups, respectively, it would be expected that consumption in the observe group would be intermediate between the two training groups. If differences in intake reflect either an effect of inhibition or approach training only, the observe group is expected to significantly differ from either the inhibition or control group, respectively.

A final aim of this study was to explore the effects of training on *healthy* food consumption.

Veling et al. (2013a) showed that when participants were trained to inhibit their responses to unhealthy foods on a go/no-go task they selected significantly fewer unhealthy snacks and significantly more healthy snacks on a hypothetical choice task (these effects were statistically significant only when comparing individuals with a high appetite or frequency of consumption). This result appears promising for the effect of training on healthy food choices. However, because participants were forced to select eight snacks from a variety of sixteen healthy and unhealthy foods, it is unclear whether this result reflects a voluntary increase in healthy food choices or an inevitable shift due to a decreased selection of unhealthy foods. To draw any firm conclusions regarding inhibitory control training and increased healthy food behaviours it would be necessary to replicate this finding with an unforced number of choices or, alternatively, to measure actual food consumption. Blackburne et al. (2016) recently provided such evidence, reporting that participants in the training condition consumed more healthy and less unhealthy food when measured with a taste test and healthy eating questionnaire. These results are only preliminary, however, because Blackburne et al. did not include an active control group.

In Study 2 we therefore measured the consumption of both healthy and unhealthy foods comparing active and control training groups. During the training tasks inhibition was mostly targeted towards unhealthy foods whereas healthy foods were paired with a response. At present it is unclear whether this training protocol would have any effect on healthy food intake. Consistent with previous findings (Blackburne et al., 2016, Veling et al., 2013a), it is possible that inhibition training may result in the increased consumption of healthy foods, potentially as unhealthy foods become less attractive (Veling et al., 2013b). As discussed above, it is also possible that consistently responding to healthy foods may prime an approach response towards these foods, thus increasing healthy food consumption (Schonberg et al., 2014; but see Lawrence et al., 2015b).

In Study 2 participants were therefore randomly assigned to one of five training conditions: participants performed an inhibition training task (stop or no-go, respectively), a control task (double-response or go, respectively), or they passively viewed the training task (observe group). Following training participants were presented with a snack buffet with eight unhealthy and healthy foods, and were invited to consume as much food as they liked. Participants were informed that the aim of the study was to measure the effect of blood glucose levels on cognitive performance. This cover story was used to justify the free-eating snack phase so that we could measure performance both at the beginning of the study, following a 3 h fast, and following food intake. We also explored the effects of stimulus-specificity in Study 2 by including both healthy and unhealthy foods that were either *old* (foods that were presented during training) or *novel* (compared to Blackburne et al.’s (2016) study which included 2 healthy and 2 unhealthy foods in the food consumption phase, of which only 1 healthy food was also presented during training). If the effects of training on behaviour depend upon specific stimulus-stop associations, the only expected difference would be for the consumption of *old* foods that were encountered during training (see Houben, 2011, Lawrence et al., 2015a, Veling et al., 2013a). Any transfer of effect to the *novel* food items, however, would suggest that training effects can be generalised to other foods.

### 3.1 Method

#### 3.1.1 Participants

One hundred and ninety-seven restrained eaters (180 females; aged 18–47, *M* = 21.64, *SE* = 0.39) were pseudo-randomly divided into the five training groups keeping age and gender evenly distributed: stop (*n* = 46, 43 females), double-response (*n* = 49, 44 females), no-go (*n* = 35, 32 females), go (*n* = 35, 32 females) and observe groups (*n* = 32, 29 females). Sample sizes were determined according to a Bayesian stopping rule for the main difference in total food consumption between the inhibition group and the respective control group; see Supplementary Information. Data collection for the observe group stopped when there was substantial evidence of a difference from at least one other group.

#### 3.1.2 Materials/measures

**Fig. 3:**
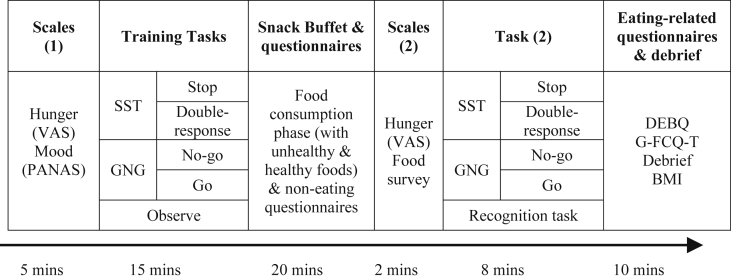
Schematic diagram of the procedure for Study 2. Following initial measures of hunger and mood, participants were randomly allocated to one of five training tasks (see Training Task for full details). They were then presented with a snack buffet with various unhealthy and healthy foods for consumption and a series of questionnaires to keep them occupied for 20 min. Participants then completed additional hunger scales, a food survey and the training task again (the observe group performed a recognition task to ensure that they were paying attention in the first training task). Participants then completed eating-related questionnaires and were debriefed.

Fig. 3 provides a schematic diagram of the procedure for Study 2.

##### 3.1.2.1 Training tasks

The training tasks lasted approximately 15 min and consisted of eight blocks of 36 trials. Participants were given a 15 s break between each block. The blocks randomly presented nine images of unhealthy foods (three images each of chocolate, crisps and biscuits), nine images of healthy foods (three images each of fruit, rice cakes and salad vegetables) and 18 filler images (clothes; three each of jeans, shirts, jumpers, socks, skirts and ties; all images were the same as those used in Lawrence et al., 2015b). One stimulus of each food type was a photographed image of the corresponding food item that was presented in the snack buffet. All images were close-up views of the food item against a white background and were matched for size and complexity.

The training tasks were similar to those presented in Study 1 with the exception that no feedback was provided for the current study, thus allowing participants in the stop group to slow down and increase their stop success rate. Following fixation (1250 ms) a stimulus appeared to the left or right of centre (1250 ms) and for no-signal trials participants were required to respond to the location as quickly and accurately as possible. The presentation of the signal and the relevant instructions for the signal trials are described below according to the training task.

###### 3.1.2.1.1 Stop-signal group

For the stop-signal group, signals were presented on 27.7% of all trials. The majority of signals were mapped onto the unhealthy foods (64 of 72; 88.89% mapping), with a minority occurring on the healthy (8 of 72; 11.11% mapping) and filler (8 of 144; 5.56% mapping) trials. These stimulus-signal mappings were used to allow for comparisons with previous stop-signal training effects (Study 1; Lawrence et al., 2015a). For the presentation of the signal the central rectangle would turn bold after a variable delay (stimulus-onset asynchrony; SOA). The SOA was initially set at 250 ms and was then continuously adjusted using a simulated tracking procedure (see Lawrence et al. (2015a) for details). Instructions for signal trials were the same as those for Study 1 (see Training Task, Study 1).

###### 3.1.2.1.2 Double-response group

The training was the same as in the stop-signal group, except that participants were instructed to make an additional response when a signal was presented (thumb response on the space bar).

###### 3.1.2.1.3 Go/no-go group

In the go/no-go training, no-go signals were consistently mapped onto the unhealthy foods (72 of 72; 100% mapping) with no signals occurring alongside the healthy food images (0 of 72; 0% mapping). These stimulus mappings are consistent with previous go/no-go training studies (Houben and Jansen, 2011, Houben and Jansen, 2015, Lawrence et al., 2015b). Filler images were inconsistently paired with a signal so that the overall rate of no-go signals was 50% (72 of 144; as in Lawrence et al., 2015b). Signals appeared as a bold rectangle that replaced the fixation rectangle and lasted for the duration of the trial (1250 ms); this meant that there was no delay between the presentation of the stimulus and the signal. Participants were instructed to withhold their response when a signal was presented.

###### 3.1.2.1.4 Go group

All trials for the go group were no-signal trials. Thus, participants were required to make a location response on every trial.

###### 3.1.2.1.5 Observe group

Participants in the observe group were presented with the same stimuli as the go group. Images were presented to the left and right hand side within a central rectangle. Participants were informed that they were to watch the stimuli, and that they needed to pay some attention because they would be asked questions at the end of the session (these questions were presented in the form of a recognition task; see Recognition Task in Supplementary Information). They were not required to make any overt motor responses to the stimuli.

##### 3.1.2.2 Snack buffet

**Fig. 4:**
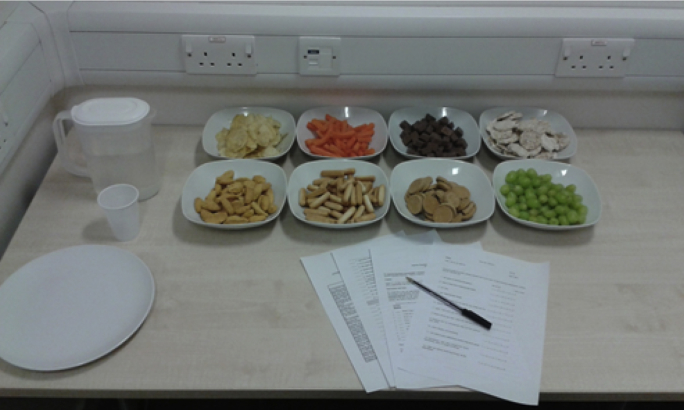
Photograph of the snack buffet layout. Participants were presented with eight bowls of healthy and unhealthy foods, water, and a series of personality questionnaires (see Questionnaires below for details). The presentation of the food items was pseudo-randomised based on healthiness and colour to minimise the effect of spatial proximity and order on consumption. Participants were left alone with the food and questionnaires for 20 min. (For interpretation of the references to colour in this figure legend, the reader is referred to the web version of this article.)

Following training participants were taken to another testing room and were presented with a snack buffet with four unhealthy foods (chocolate, crisps, biscuits and cheese bites) and four healthy food items (grapes, carrots, rice cakes, breadsticks; see Fig. 4; see Supplementary Table 6 for weight and nutritional information). The presentation of the food bowls was pseudo-randomised across participants to ensure that there was no bias in consumption based on the spatial proximity of the food item. During the snacking phase participants were asked to fill out a battery of non-eating-related personality questionnaires (see Questionnaires below) and were instructed to eat as much food as they liked but to ensure that they were not feeling hungry (in order to replenish their glucose levels, consistent with the cover story) when the experimenter returned after 20 min. Unknown to the participants, all food items were weighed before and after the snack buffet to measure calorie consumption.

##### 3.1.2.3 Questionnaires

During the snack buffet participants were provided with a battery of personality questionnaires (see footnote 2). The purpose of these questionnaires was to keep the participant occupied for the duration of the snacking phase. After 20 min participants were taken back to the original testing room. They completed three hunger measures (100 mm visual analogue scales to measure hunger, fullness and desire to eat; Flint et al., 2000, Yeomans, 2000) along with a brief survey regarding the food items presented in the snack buffet. This questionnaire asked participants how often they normally consumed the food items (7 point Likert scale from 1 “Never” to 7 “Daily”), whether they consumed each of the items during the snack phase (this was to reduce the likelihood of participants believing that their food consumption was being measured and to help maintain a naïve sample), and if so how much they liked the taste of the item (10 point Likert scale from 1 “I didn't like the taste at all” to 10 “I liked the taste very much”).

#### 3.1.3 Procedure

The procedure for Study 2 is outlined in Fig. 3. As with Study 1, all participants were asked to refrain from eating for 3 h prior to the study and all testing took place between the hours of 12–7 pm. After giving consent, participants answered initial hunger and mood questionnaires (see Procedure, Study 1). They then completed the training task before being taken to the snack buffet. After 20 min the experimenter brought them back to the original testing room to answer the hunger scales and food survey (see Questionnaires above), and to complete the training task again. This task was identical to the initial training task with the exception that it only lasted 10 min (4 blocks of 36 trials). The only purpose of this task was to make the cover story (i.e. cognitive testing after replenishing glucose levels) plausible. The observe group completed a recognition task consistent with the cover story for that group. After finishing this task, participants completed two eating-related questionnaires (see footnote 2), they were debriefed (see Supplementary Information for debrief questions and statistical analyses) and their height and weight was measured.

### 3.2 Statistical analyses

Statistical analyses for Study 2 are broadly consistent with those presented in Study 1; here we focus on the additional analyses for the present study.

To provide a partial replication of previous studies showing effects of either stop or go/no-go training on food consumption (Houben, 2011, Houben and Jansen, 2011, Houben and Jansen, 2015, Lawrence et al., 2015a, Veling et al., 2011) we first compared the effectiveness of each inhibition task relative to the control task within that training protocol (i.e. stop *vs*. double-response and no-go *vs*. go). These analyses are presented in the Supplementary Information. The groups were then analysed together, including the additional observe group, to allow for a direct statistical comparison.

Analysis of group demographic, state and trait variables and training data was identical to that in Study 1 (see Supplementary Information for details). Performance data for the recognition task was also analysed to ensure that participants in the observe group were paying attention during the ‘training’ task. Participants performed this task well (accuracy: *M* = 91.41%; *SE* = 0.74); no exclusions were made according to the pre-set criterion of 70% accuracy. Following exclusions there was a final sample size of 43 participants in the stop group, 42 participants in the double-response group, 34 participants in the no-go group, 32 participants in go group and 32 participants in the observe group.

Food intake was analysed as a function of food type and food novelty by calculating the *mean* calorie value for each food category; the total calories for each food category was divided by the number of foods in that category. For example, for unhealthy old foods there were three different foods (chocolate, crisps and biscuits) but for unhealthy new foods there was only one food presented (cheese bites); calorie consumption for unhealthy old foods was therefore divided by three to calculate the *mean* intake for that category. The consumption of healthy foods was also analysed in grams to avoid potential floor effects (see Supplementary Information).

### 3.3 Results

#### 3.3.1 Group differences

Training groups were well matched for all measures (see Supplementary Information for details).

#### 3.3.2 Consumption data analysis

Results of the separate analyses (for stop *vs*. double-response training and no-go *vs*. go training) revealed that participants in the stop group consumed 18% fewer calories than those in the double-response group, whereas participants in the no-go group consumed 32% fewer calories than those in the go group. These findings were statistically significant (both *p*s < 0.04, both B > 3.73 in favour of H1; see Supplementary Information) and are consistent with previous research showing a 30–50% reduction in food consumption following inhibition training (Houben and Jansen, 2011, Houben and Jansen, 2015, Houben, 2011, Lawrence et al., 2015b, Veling et al., 2011).

**Fig. 5:**
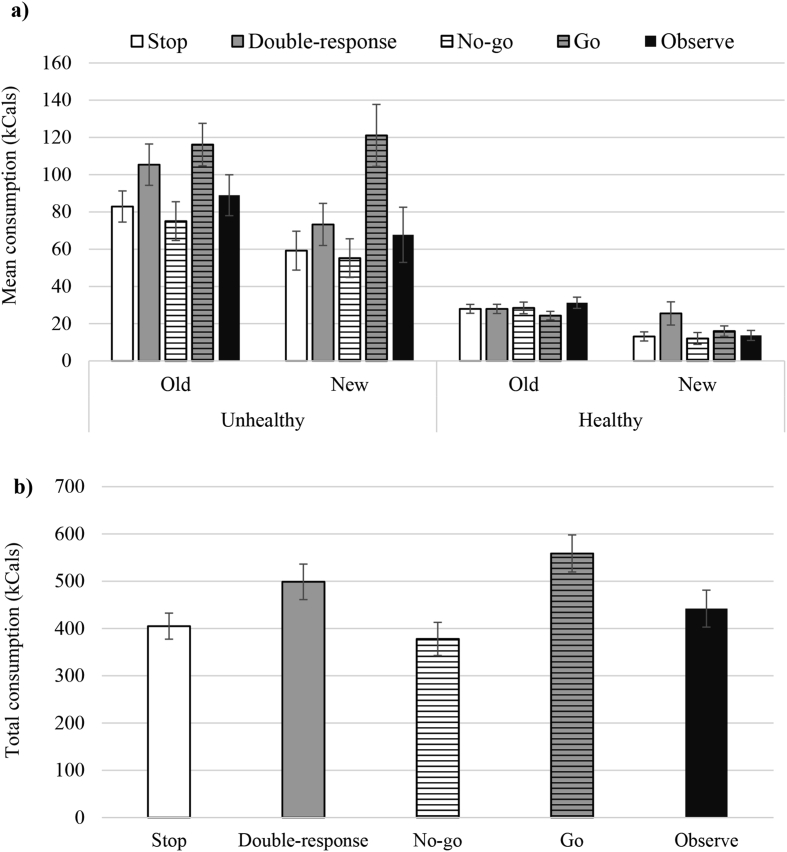
**a)** Mean calorie consumption (total calories/number of foods per category) as a function of training condition for the unhealthy and healthy foods that were presented in both the training and the snack buffet (old) and those that were presented in the snack buffet only (new). **b)** Total calorie intake as a function of training condition. Error bars show ±1SE.

A 5 x 2 x 2 mixed ANOVA (between-subjects factor: *training condition* [stop, double-response, no-go, go, observe]; within-subjects factors: *food type* [healthy, unhealthy] and *food novelty* [old, new]) for the complete analysis revealed a significant main effect of training condition (*p* < 0.001, *ƞ*_p_^2^ = 0.11) and a significant interaction between training condition and food type (*p* < 0.001, *ƞ*_p_^2^ = 0.11; see Fig. 5). The interaction showed that the effect of training was specific to the consumption of unhealthy foods (*F* (4,178) = 5.84, *p* < 0.001, *ƞ*_p_^2^ = 0.12). There was no effect of training on healthy food intake measured in either calories (*F* (4,178) = 1.56, *p* = 0.19, *ƞ*_p_^2^ = 0.03) or grams (*p* = 0.33; see Supplementary Information). After correcting for multiple significance tests (α/10 = 0.005), pairwise comparisons for unhealthy calorie intake showed that consumption in the go group was significantly greater than consumption for the no-go (*p* < 0.001; *d* = 1.11), stop (*p* < 0.001; *d* = 0.97) and observe (*p* = 0.002; *d* = 0.81) groups. The difference between double-response and stop groups was no longer statistically significant following correction for multiple comparisons (*p* = 0.1; *d* = 0.36). No other comparisons reached statistical significance (all *p*s > 0.015, all *d*s < 0.58). The three-way interaction between training condition, food type and food novelty was also non-significant (*p* = 0.52, *ƞ*_p_^2^ = 0.02).

Bayes factors for comparisons between the observe group and other training groups revealed substantial evidence for H1 in the comparison against the go group (B = 3.62; mean difference = 116.6, SE of the difference = 55.35; JZS Bayes = 1.63), however, all other comparisons were inconclusive (vs stop: B = 1.17; JZS = 0.32; vs double-response: B = 1.42; JZS = 0.38; vs no-go: B = 1.65; JZS = 0.48).^5^

### 3.4 Discussion

The results of Study 2 support previous research showing that, relative to the ‘go’ control groups, both stop training and no-go training were effective at reducing unhealthy food consumption (see Supplementary Information; Houben, 2011, Houben and Jansen, 2011, Houben and Jansen, 2015, Lawrence et al., 2015a, Veling et al., 2011). There were no effects of training on healthy food intake, however. Furthermore, our findings are consistent with three recent meta-analyses indicating greater effects of go/no-go training compared to stop-signal training (Allom et al., 2015, Jones et al., 2016; Turton et al., 2016). However, the comparison with the observe group suggests that these training effects were partly due to increased consumption in the go-control groups (in particular the go group) whereas evidence for reduced consumption in the inhibition groups was inconclusive. The results of the overall analysis, with all five training groups, revealed a significant effect of training on the consumption of unhealthy foods only, and in particular, increased consumption in the go group relative to both inhibition groups and the observe group. The two potential explanations for these findings are that go-training increased unhealthy food intake, and/or the observe task decreased intake. Both of these explanations have some supporting evidence and are outlined in detail in the General Discussion below. These explanations are not mutually exclusive so we suggest that the findings of Study 2 result from a combination of both effects.

## 4 General discussion

Across two studies we investigated the effects of response inhibition training on food consumption and explored the roles of stimulus devaluation, stimulus-specific associations and training protocols. In Study 1 we showed no effect of stop-signal training on either implicit attitudes or chocolate consumption. It is possible that the intermediate attitude task dampened any effects of training on food consumption, but critically we also argue that these null results are due to the extra demands placed on action cancellation during this stop training task. In agreement with the conclusions of Jones et al. (2016) we believe that the low rate of overall stop success hindered the development of strong stimulus-stop associations (Verbruggen and Logan, 2008a, Verbruggen and Logan, 2008b, Verbruggen et al., 2014b). To examine whether inhibition training had an effect on reduced food intake when the rate of successful inhibition was higher, in Study 2 we allowed people to slow down in the stop task (thus increasing stop success rate) and compared training on the stop-signal task with training on the go/no-go task. The results of Study 2 revealed differences between the stop-signal and no-go training groups and their corresponding control conditions (i.e. the double-response group and the go group, respectively, see Supplementary Information), with larger effects following no-go training. These findings are consistent with three recent meta-analyses showing greater effects of go/no-go than stop-signal training on food- and alcohol-related behaviour change (Allom et al., 2015, Jones et al., 2016; Turton et al., 2016), and support the idea that the rate of successful inhibitions may moderate the effects of training on behaviour (Jones et al., 2016). However, from the evidence to date it is unclear to what extent the increased successful stop rate on the go/no-go task is due to the simultaneous presentation of the stimulus and stop cue (and the process of action restraint, compared to action cancellation; Eagle et al., 2008, Schachar et al., 2007), the more consistent stimulus-signal mapping or both. Future research could explore the effect of such training parameters on behaviour and investigate the role of learning stimulus-stop associations and automatic, compared to controlled inhibition (McLaren and Verbruggen, 2016, Spierer et al., 2013, Verbruggen et al., 2014a, Verbruggen et al., 2014b). The same is also true for other training parameters that vary across different studies (including the number and proportion of stop-stimulus pairings, number of sessions, number of trials, and the type and number of stimulus categories used).

Results from a passive observe group, however, suggest that effects of inhibition training on behaviour observed in the lab may be partly driven by stimulus-*go* associations increasing food consumption in the ‘go’ control groups. These results lend support to previous studies that have found effects of go or ‘approach’ training on behaviour (Kemps et al., 2013, Schonberg et al., 2014). Just as it has been argued that inhibition training may reduce food consumption by encouraging the development of stimulus-stop associations and activating an aversive centre, it is also possible that go training may encourage the development of stimulus-go associations and activation of an appetitive centre (McLaren and Verbruggen, 2016, Verbruggen et al., 2014b, Verbruggen et al., 2014a). Indeed recent findings suggest that in some experiments, go-associated food stimuli show an increase in valuation (relative to untrained food stimuli) after go/no-go training (Chen, Veling, Dijksterhuis, & Holland, 2016). This go-training effect may have contributed to effects of food inhibition training shown in previous studies (e.g. Houben and Jansen, 2011, Houben and Jansen, 2015, Houben, 2011, Houben et al., 2012a, Houben et al., 2011, Lawrence et al., 2015a, Veling et al., 2011) and also potentially applies to other behavioural interventions that have trained approach-avoidance tendencies or attentional biases (e.g. Hardman et al., 2013, Kemps et al., 2013, Werthmann et al., 2014, Wiers et al., 2010). Intriguingly however, we did not find that double-response training resulted in increased food consumption compared to the observe group. This finding may be explained by the additional elements of the double-response task, such as visual detection and action updating, which may have primed disinhibition to a lesser extent than the single-response go task – possibly by engaging neural networks that are also involved in the inhibition of responses (although there was no difference in GoRTs between double-response and go groups in the current study; see Aron et al., 2014, Verbruggen et al., 2010). However, because the double-response group did not significantly differ from either observe or go groups this suggestion is only tentative and requires further investigation.

The other explanation for the increased consumption in the go group relative to the observe group (and the two inhibition groups) is that the observe group in Study 2 is also not a true baseline. Rather, the observation of food stimuli without responding could be argued to be another form of inhibition training. Such a prolonged period of food cue exposure with response prevention could be considered highly atypical whereas approaching pleasant foods may best represent how we naturally interact with these foods. A recent study provides some support for this idea – participants undergoing a similar observe-control condition showed significantly greater devaluation of ‘go’ food stimuli than participants who had to respond to these foods in a go/no-go task (Chen et al., 2016). This occurred in the absence of increased valuation of go-images in some experiments. It may therefore be very difficult to find a truly neutral control condition that matches for food cue exposure in studies of inhibition training. Another potential control task is to pair target images equally with a response and with inhibition (i.e. 50% mapping). Houben (2011) and Houben and Jansen (2011) have used this condition, reporting that it serves as a baseline “without inducing impulsivity or inhibition” (p.386, Houben, 2011). However, there is evidence in both of these studies that participants consumed more calories in the control condition (i.e. the inconsistent mapping) than in the impulsivity condition (i.e. the always-go mapping). These unexpected results may be explained by findings indicating that associative uncertainty can increase attention to, incentive salience for, and responding towards conditioned stimuli (e.g. Anselme et al., 2013, Pearce and Hall, 1980). Perhaps the most conservative control condition is one that requires the inhibition of responses to non-food stimuli (Lawrence et al., 2015b, Veling et al., 2014) – although, without any exposure to food stimuli it is possible that using this task underestimates any effects of inhibition training.

Perhaps the most convincing evidence to date for the validity of inhibition training are the positive effects on weight loss from pre-to post-training relative to a control group that had no exposure to food cues (i.e. they received inhibition training for non-food stimuli and received no food-go trials; Lawrence et al., 2015b, Veling et al., 2014). It is difficult to argue that these results could be driven by increased food intake in the control group, who did not show any changes in weight. Together these results imply that inhibition training may be an effective intervention for reducing food consumption and aiding weight loss, particularly when tasks feature strong stimulus-stop associations. However, the results we present here highlight the importance of the training protocol and the potential confounds associated with some ‘control’ groups. We suggest that researchers carefully consider the training and control tasks used, as well as the dependent measures employed in future research. Perhaps the easiest and most valid way to overcome this issue is to assess the effectiveness of such interventions on actual weight loss.

## Footnotes

1 Words were selected and matched as closely as possible for scores on concreteness, familiarity, imageability, number of syllables, verbal frequency (Brown verbal frequency) and written frequency (Kucera-Frances and Thorndike-Lorge written frequency measures) according to the MRC Psycholinguistic Database: http://websites.psychology.uwa.edu.au/school/MRCDatabase/uwa_mrc.htm.

2 Filler questionnaires included the following: The Big Five Inventory (BFI-44; John, Naumann & Soto, 2008), the Brief Self Control Scale (BSCS; Tangney, Baumeister & Boone, 2004), the Emotion Regulation Questionnaire (ERQ; Gross & John, 2003), the UPPS impulsive behaviour scale (Whiteside & Lynam, 2001), the Attentional Control Questionnaire (ACQ; Derryberry & Reed, 2002) and the Mood and Anxiety Symptom Questionnaire (MASQ-62; Watson et al., 1995). After 20 min when the questionnaires and foods were collected, participants were asked to complete the Dutch Eating Behaviour Questionnaire (DEBQ; Van Strien, Frijters, Bergers & Defares, 1986) and the General Food Craving Questionnaire – Trait version (G-FCQ-T; Cepeda-Benito, Gleaves, Williams & Erath, 2000; Nijs, Franken & Muris, 2007). These questionnaires were included for the purpose of another study (Adams, Lawrence, Verbruggen & Chambers, in preparation) to examine test-retest reliability for the DEBQRE following inhibition training and to explore differences in food craving between different measures of dietary restraint.

3 (http://dx.doi.org/10.6084/m9.figshare.1619681).

4 There were also no significant correlations between positive or negative SC-IAT scores and chocolate consumption for either training group (all *r*s < 0.13, all *p*s > 0.31).

5 Bayes factors for Study 2 calculated using the expected difference score (see Supplementary Information) must be interpreted with caution as they are likely to be overestimated. The expected difference score was based on previous studies that have typically included 1–3 foods in a bogus taste test, whereas in this study 8 foods were presented and participants were asked to have as much food as they liked as long as they were no longer hungry after 20 min. It is possible therefore that participants in the present study would consume more calories than participants in previous studies as increased food variety has been associated with increased food intake (Guerrieri, Nederkoorn & Jansen, 2008; but see Guerrieri et al., 2007, Guerrieri et al., 2008). Indeed, results for total calorie consumption (see Fig. 5b) support this position with participants consuming a greater amount of food (380 + kcal), compared to previous studies (Houben and Jansen, 2011, Houben and Jansen, 2015, Houben, 2011, Lawrence et al., 2015a).
