# Supplementary material for: Training response inhibition to reduce food consumption: Mechanisms, stimulus specificity and appropriate training protocols
Source: Appetite. 2017 Feb 1;109:11–23. doi: 10.1016/j.appet.2016.11.014 (PMC5240656; doi:10.1016/j.appet.2016.11.014)
Supplement: Online data [file mmc1.docx]

**Supplementary Information:**

**Training response inhibition to reduce food consumption: Mechanisms, stimulus specificity and appropriate training protocols**

**Participant Debrief**

During the debrief, participants were probed for knowledge of the study aims and stimulus mappings with open-ended, funnelled questions. Specifically, they were asked a) whether they noticed anything in particular in the training task, b) whether they noticed anything about when they had to stop or make a double response, c) whether they thought the signals were distributed evenly, randomly or were grouped in any way. Although previous findings are mixed, some studies have reported training effects only for participants who were aware of the experimental contingencies (Attwood, O’Sullivan, Leonards, Mackintosh & Munafò, 2008; Field et al., 2007; Field, Duka, Tyler & Schoenmakers, 2009; Hogarth, Dickinson, Hutton, Bamborough & Duka, 2006; Kakoschke, Kemps & Tiggemann, 2014). Furthermore, a moderating role of awareness also has theoretical implications for whether training effects are due to controlled or automatic inhibition (see Best, Lawrence, Logan, McLaren, Verbruggen, 2015; Verbruggen et al., 2014b) and could indicate that training effects are due to demand characteristics (Boot, Simons, Stothart & Stutts, 2013). Participants were also asked whether they thought that performing the first computer task had any influence on the second computer task, the taste test or questionnaires. They were then asked if they had participated in any related studies, if they were currently dieting, if they had any history of eating disorders, and at what time they last ate to allow for exclusions based on these criteria. Checks for these factors were made prior to testing but were asked again during the debrief for clarification.

**Statistical Analyses, Study 1**

The following demographic, state and trait variables were analysed to ensure that there were no statistically significant differences between training groups: gender ratio, age, BMI, chocolate craving (ACQC), dietary restraint (RS), hours since last food consumption, hunger (VAS measures), mood (PANAS), food palatability ratings and food consumption ratings.

Training data was analysed to guide participant exclusions and all criteria were decided prior to hypothesis testing. The three variables of interest were the percentage of erroneous responses on signal trials (failed inhibition for the stop group and failure to execute both responses in the double-response group; in the double-response group, these errors also included trials in which an incorrect location response was made), the mean reaction time for no-signal trials (GoRT) and the percentage of errors on no-signal trials (including incorrect location responses and missed responses). Participants were excluded if their percentage errors on signal trials was >3SDs from the group mean, their GoRT was >3SDs from the group mean or their accuracy on no-signal trials was <85%. Details of participant exclusions are provided in Supplementary Table 1 below.

**Supplementary Table 1.** Reasons for participant exclusions according to training condition.

| Study | 1 | 1 | 2 | 2 | 2 | 2 | 2 |
| --- | --- | --- | --- | --- | --- | --- | --- |
| Group | Stop | Double-response | Stop | Double-response | No-go | Go | Observe |
| Reason for exclusion | |  |  |  |  |  |  |
| % errors on signal trials  (>3 SDs group mean) | 0 | 0 | 1 | 2 | 0 | 0 | 0 |
| GoRT on no-signal trials (>3SDs group mean) | 0 | 2 | 0 | 0 | 0 | 0 | 0 |
| % errors no-signal trials  (>85% accuracy) | 1 | 1 | 0 | 1 | 0 | 2 | 0 |
| Already participated in a similar study | 0 | 0 | 2 | 1 | 0 | 0 | 0 |
| Dieting or eating disorder | 0 | 0 | 0 | 2 | 1 | 1 | 0 |
| Guessed aim | 0 | 0 | 0 | 1 | 0 | 0 | 0 |
| **Total** | **1** | **3** | **3** | **7** | **1** | **3** | **0** |

Two unipolar SC-IATs were used to measure the second dependent variable of implicit attitudes. IAT effects were calculated using the scoring algorithm of Karpinsky and Steinman (2006), which is modelled on the *D*-score algorithm for the IAT (Greenwald, Nosek & Banaji, 2003). All data from practice blocks were discarded as planned, along with non-responses and responses in experimental blocks that were less than 350 ms or more than 1000 ms. Errors were replaced with the block mean plus an error penalty of 600 ms (the higher penalty of 600 ms was used in accordance with Houben et al*.*, 2010, 2011, 2012a, 2012c). The average response time in the target pairing block was subtracted from the average response time in the neutral pairing block and the result was divided by the standard deviation of all correct response times in both blocks. Higher scores therefore indicate a faster performance on the target pairing block relative to the neutral pairing block, whereas negative scores would reflect the reverse.

For the primary dependent variable of food consumption, outliers were considered as values >3SDs from the group mean for each food type separately. Outlier values were replaced with the nearest non-outlier value +1; this method reduces the impact of a univariate outlier while maintaining the score as the most deviant (Tabachnick & Fidell, 2007).

A sensitivity analysis, using G*Power (Faul, Erdfelder, Lang & Buchner, 2007), for the main interaction between training condition and food type revealed that our sample had 80% power to detect a minimum effect size of *f*=0.21 (*ƞ*_p_²=0.04) for the main effect of training condition and 80% power to detect an effect size *f*=0.11 (*ƞ*_p_²=0.01) for the interaction [α=0.05, number of groups =2, number of repetitions =2, correlation among repeated measures =0.59 (calculated post hoc based on the correlation between chocolate and crisp consumption), non-sphericity correction =1]^[[1]](#footnote-1)^.

All results are reported with unadjusted significance values; corrections for multiple comparisons were calculated for all within-test analyses and are only reported where these corrections changed the interpretation of an analysis from statistically significant to non-significant. All statistical analyses were performed with SPSS.

Bayes factors were also calculated for two reasons. Firstly, Bayesian hypothesis testing allows for a flexible stopping rule on data collection without correcting for the elevation of Type I error, as would be required under a frequentist approach (Dienes, 2011, 2014). Secondly, unlike null hypothesis significance testing, Bayes factors can be used to interpret the evidential value of negative findings (Dienes, 2011, 2014; Rouder, Speckman, Sun, Morey & Iverson, 2009).

To calculate an expected difference for total calorie intake between the inhibition group and the control group (the Bayesian prior), available results were entered into a Bayesian meta-analysis (Dienes, 2014^[[2]](#footnote-2)^). These results were limited to studies that compared the effect of a single session of food-related inhibition and control training on food consumption in a sample who were believed to show poor inhibitory control towards food at the outset. They were obtained from Houben (2011; the difference in consumption between the go and stop foods for individuals with low inhibitory control; 25.59 kCals), Veling et al*.* (2011; Study 2; the difference in consumption between control and no-go groups for chronic dieters; 179.46 kCals), Lawrence et al*.* (2015a; the difference in total consumption between double-response and stop groups for participants with high dietary restraint; 60.09 and 142.01 kCals for Studies 1 & 2, respectively) and Houben and Jansen (2015; the difference in consumption between go and no-go groups; 46.93 kCals). The results of this meta-analysis produced an estimated difference of 48.51 kCals. To calculate the Bayes factor for food consumption, following Dienes (2011, 2014), this value was entered as the standard deviation in a half-normal distribution with a mean value of 0; a half-normal distribution was used as smaller effects were considered more probable than larger effects. For the sample mean, a between-subjects t-test was performed for the effect of training condition (stop or double-response) on total food consumption. The mean difference and standard error of the difference for this comparison were entered into Dienes’ online calculator^[[3]](#footnote-3)^. The result of this calculation was therefore used to guide data collection; data collection was terminated when the Bayes factor provided substantial evidence for either the alternative hypothesis (B>3; stop training reduces food intake) or the null hypothesis (B<0.33; stop training does not reduce food intake). If the Bayes factor had remained inconclusive a maximum sample size was set at 172 participants; 172 participants would achieve 90% power to detect a medium effect size of Cohen’s dz=0.5 using a two-tailed independent t-test (a comparison between active and control training conditions for the effect on overall food consumption) with an alpha level of 0.05 (sample size acquired using G*Power; Faul et al., 2007).

For the two unipolar SC-IATs in Study 1, there was no available data to inform the expected difference between training groups. We therefore calculated Bayes factors using the default JZS prior (Rouder et al., 2009). The JZS prior is a non-informative objective prior that minimises assumptions regarding expected effect size. Bayes factors using the JZS prior were calculated by entering *t* statistics and sample sizes into using Rouder et al.’s online calculator (*r* was set a priori to the default value, *r* = 0.707)^[[4]](#footnote-4)^. Results in favour of the alternative hypothesis were inversed (1/B) for ease of interpretation (such that B>3 suggests ‘substantial’ evidence for the alternative hypothesis and B< 0.33 indicates ‘substantial’ evidence for the null hypothesis; Dienes, 2011, 2014).

**Additional Analyses, Study 1**

**Group Differences**

The two training groups were well-matched: there were no significant differences in age, BMI, chocolate craving (ACQC) dietary restraint (RS), and hours since food consumption (all *p*s>0.2, all *d*s<0.22; see Supplementary Table 2). The gender ratio was also similar for both groups with five males in the stop group and 4 males in the double-response group; due to the small number of males in each condition, we did not conduct a Chi square analysis. There were also no significant differences in state measures of hunger (VAS measures) and mood (PANAS; all *p*s>0.27, all *d*s<0.19). Participants also reported similar levels of palatability and frequency of consumption for both foods in the taste test (all *p*s>0.16, all *d*s<0.24).

**Supplementary Table 2.** Group characteristics and between-group significance tests for Study 1 (SE within parentheses).

|  | Stop  (*n*=70) | Double-response  (*n*=69) | *t* = | *p =* |
| --- | --- | --- | --- | --- |
| Gender (% female) | 92.9 | 94.2 |  |  |
| Age | 23.1 (1.01) | 22.03 (0.73) | 0.86 | 0.39 |
| BMI | 24.32 (0.53)^1^ | 24.75 (0.56)^2^ | -0.55 | 0.58 |
| ACQC | 18.9 (0.7) | 18.29 (0.65) | 0.64 | 0.53 |
| RS | 18.89 (0.3) | 19.58 (0.44) | -1.30 | 0.20 |
| Hours since food | 5.06 (0.37) | 5.37 (0.46) | -0.54 | 0.59 |
| Hunger | 5.47 (0.21) | 5.4 (0.21) | 0.23 | 0.82 |
| Fullness | 1.49 (0.17) | 1.52 (0.19) | -0.13 | 0.90 |
| Desire to eat | 6.14 (0.26) | 5.74 (0.25) | 1.11 | 0.27 |
| Positive affect | 25.35 (0.8)^2^ | 25.43 (0.71) | -0.08 | 0.93 |
| Negative affect | 13.01 (0.45)^2^ | 13.14 (0.48) | -0.20 | 0.84 |
| Chocolate palatability | 8.85 (0.15)^1^ | 8.51 (0.19)^1^ | 1.40 | 0.16 |
| Chocolate consumption freq. | 4.79 (0.07) | 4.67 (0.09) | 1.05 | 0.30 |
| Crisp palatability | 7.15 (0.26)^1^ | 7.34 (0.23)^1^ | -0.53 | 0.60 |
| Crisp consumption freq. | 3.53 (0.17) | 3.71 (0.14) | -0.81 | 0.42 |
| FCQ-T | 4.1 (0.11)^4^ | 3.93 (0.11)^8^ | 1.10 | 0.27 |

Note. Superscript denotes the number of participants missing for that variable. ACQC= Attitudes to Chocolate Craving subscale; RS=Restraint Scale; BMI=body mass index; FCQ-T= Food Craving Questionnaire – Trait.

**Training Data Analysis**

The training data show that, on average, participants in the stop group correctly inhibited their responses on 54.79% of signal trials (*SE*=0.78); at 50% the stop and go processes are theorised to be at a point of maximum competition (Logan, Schachar & Tannock, 1997). The higher probability of stopping for chocolate images (69.76%, SE =1.28) indicates that participants learned the chocolate-stop associations to a certain extent. On average, participants in the double-response group responded correctly on 95.54 % (*SE*=0.48) of signal trials, demonstrating that they performed the task correctly. For the GoRT there was a significant difference between the two groups, with participants in the stop group (*M*=522.65, *SE =* 12.06) responding significantly slower than participants in the double-response group (*M*=396.72, *SE =*7.12; *p*<0.001, *d*=1.52). This suggests that participants increased response thresholds when they expected a stop signal (Verbruggen & Logan, 2009). Performance accuracy on no-signal trials was high and errors did not differ significantly between the stop (*M*= 1.35, *SE =*0.24) and double-response groups (*M*=1.64, *SE =*0.23; *p*=0.38, *d*=0.15).

**Unipolar, SC-IAT Data Analysis**

For the positive/pleasant SC-IAT, one-sample t-tests (test value of 0) revealed statistically significant positive attitudes towards chocolate for the stop (*M*=0.49; *SE*=0.06; *t*(69)=7.53, *p*<0.001, *dz*=0.91) and double-response (*M*=0.36; *SE*=0.06; *t*(68)=6.33, *p*<0.001, *dz*=0.76) groups. For the negative/unpleasant SC-IAT, neither the stop (*M*=-0.01; *SE*=0.06; *t*(68)=-0.21, *p*=0.84, *dz*=0.02) nor the double-response group (*M* =-0.07; *SE*=0.05; *t*(68)=-1.32, *p*=0.19, *dz*=0.16) showed a significant attitude towards chocolate (see Supplementary Figure 1). Contrary to expectation, the mean score on the positive SC-IAT was greater for the stop group compared to the double-response group, whereas, the scores on the negative SC-IAT were in the expected direction.

The 2x2 mixed ANOVA [between-subjects factor: *training condition* (stop or double-response); within-subjects factor: *SC-IAT* (positive or negative)], however, revealed no significant main effect of training condition (*F*(1, 136)=2.27, *p*=0.13, *ƞ*²_p_ =0.02), and importantly, no significant interaction between training condition and SC-IAT (*F*(1, 136)=0.36, *p*=0.55, *ƞ*²_p_=0.003). There was a significant main effect of SC-IAT with a positive score on the positive/pleasant SC-IAT (*M*=0.42; *SE*=0.04) and a negative score on the negative/unpleasant SC-IAT (*M*=-0.04; *SE*=0.04; *F*(1, 136)=67.12, *p*<0.001, *ƞ*²_p_ =0.33).

For the positive SC-IAT the JZS Bayes factor was 0.53. Although this is only weak evidence in favour of the H0, the results are in the opposite direction to that expected. The Bayes factor for the negative SC-IAT was 0.23, providing substantial evidence in favour of H0 over H1.

**Supplementary Figure 1.** Mean bias scores for the positive (pleasant) and negative (unpleasant) SC-IATs (D600 scoring algorithm) according to training condition. Positive values indicate a faster association for target category pairing trials (chocolate and pleasant words on the positive SC-IAT and chocolate and unpleasant words on the negative SC-IAT) whereas negative values indicate a faster association in the neutral category pairing trials (chocolate and neutral words). Error bars show ±1 SE.

**Supplementary Table 3.** Consumption data and statistical comparisons for Study 1 (SE within parentheses).

|  | Stop  (*n*=70) | Double-response  (*n*=69) | *F*= | *p=* | *ƞ*²_p_ *=* |
| --- | --- | --- | --- | --- | --- |
| Chocolate | 238.45 (17.97) | 197.15 (19.91) |  |  |  |
| Crisps | 160.96 (13.58) | 144.91 (13.44) |  |  |  |
| Total | 399.41(27.75) | 333.28 (27.52) |  |  |  |
| Condition |  |  | 1.95 | 0.16 | 0.01 |
| Food |  |  | 34.83 | <0.001 | 0.2 |
| Condition*Food |  |  | 1.32 | 0.25 | 0.01 |

**Debrief Analysis**

During the debrief, participants were probed for awareness of the stimulus mappings using funnelled questions. If they indicated awareness that the majority of signals were mapped onto the chocolate stimuli they were considered ‘aware’, if they mentioned that signals were mapped onto food in general they were considered ‘partially aware’, and if they reported no associations they were considered ‘not aware’. Across both training groups, 50% of participants noticed that signals were paired with chocolate, 11.6% reported that signals were paired with food in general and 38.4% did not report any consistent stimulus-signal associations. These values were similar across the two training conditions (see Supplementary Table 4 for all debrief frequency data). For the chi-square test the aware and partially aware participants were collapsed into one group due to the small number of participants who were partially aware. The test revealed that the differences between the two groups were not statistically significant (χ ^2^(1)=1.02, *p*=0.31, *ϕ*=0.09). Furthermore, a mixed 2x2x2 ANOVA with *training condition* (stop or double-response) and *awareness* (aware or not aware) as between-subjects factors and *food type* (chocolate and crisps) as a within subjects factor revealed that awareness of stimulus mappings did not have any discernible effect on food consumption; the main effect of awareness and interactions with awareness were not statistically significant (all *F*s<1.74; all *p*s>0.19; all *ƞ*²_p_s<0.013).

Importantly, no participants correctly guessed the aim of the study and no participants mentioned an awareness that their food intake was being measured (an awareness that food intake is being monitored can produce floor effects; see Robinson, Kersbergen, Brunstrom & Field, 2014; Roth, Herman, Polivy & Pliner, 2001). When asked whether they thought that the training had any influence on the food they had, the majority of participants answered that the training had no effect (78.3%) and a minority of participants reported that the training made them hungry or desire food (21.7%). These values were similar for the stop and double-response groups: 24.3% and 19.1%, respectively, reported increased hunger or desire to eat. A chi-square test revealed no statistically significant difference between groups (*χ^2^*(1)=0.54, *p*=0.46, *ϕ*=0.06).

**Supplementary Table 4.** Results of the debrief analysis, Study 1. Results show frequencies of responses across training conditions.

|  | Stop  (*n*=70) | Double-response  (*n*=69) |
| --- | --- | --- |
| Awareness of stimulus mappings |  |  |
| Aware | 40 (57.1%) | 29 (42.6%) |
| Partially aware | 6 (8.6%) | 10 (14.7%) |
| Not Aware | 24 (34.3%) | 29 (42.6%) |
| Influence of training |  |  |
| No effect | 53 (75.7%) | 55 (80.9%) |
| Hunger or desire food | 17 (24.3%) | 13 (19.1%) |

**Study 2**

**Sensitivity Analysis**

For Study 2, a sensitivity analysis (using G*Power; Faul et al., 2007) of the main interaction between training condition and food type revealed that the ‘stop-signal vs. double-response’ comparison had 80% power to detect a minimum effect size of *f*=0.22 (*ƞ*_p_²=0.05) for both the main effect of training condition and the interaction (α=0.05, number of groups =2, number of repetitions =2, correlation among repeated measures = -0.01 (correlation between unhealthy and healthy food consumption), non-sphericity correction =1). The ‘go/no-go vs. go’ comparison had 80% power to detect a minimum effect size of *f*=0.26 (*ƞ*_p_²=0.06) for the main effect of training condition and *f*=0.23 (*ƞ*_p_²=0.05) for the interaction (α=0.05, number of groups =2, number of repetitions =2, correlation among repeated measures =0.1, non-sphericity correction =1). For the full sample, with all five training conditions, there was 80% power to detect a minimum effect size of *f*=0.18 (*ƞ*_p_²=0.03) for the main effect of training condition and the interaction (α=0.05, number of groups =5, number of repetitions =2, correlation among repeated measures = 0.005, non-sphericity correction =1).

**Recognition Task**

The recognition task was presented to the observe group only to ensure that they did indeed observe the training task; the task consisted of one block of 72 trials. Each trial presented a central image that was either an image repeated from the training phase or a new image. All 36 images from the training task were used, and for the remaining 36 trials: 12 were completely novel objects that were not representative of the images presented in the training phase (e.g. plants and household objects); 12 were relatively similar images of foods and clothes (for example a different type of fruit) and 12 were very similar objects of foods and clothes (for example a different image of an apple). The relatively and very similar items were included to ensure a certain degree of difficulty. Each stimulus was presented until a response was given or for a maximum of eight seconds. For each trial participants had to indicate whether or not they recognised the image from the training task (responding ‘Old’ or ‘New’ with the ‘J’ and ‘K’ buttons, respectively, on a standard keyboard). They were instructed to respond as quickly and accurately as possible; if they were unsure they were told to make their best guess.

**Supplementary Table 5.** Nutritional information and weights for the unhealthy and healthy foods presented in the snack buffet.

|  | | Weight  provided (g) | kCals  per 100g | Fat  per 100g |
| --- | --- | --- | --- | --- |
| Unhealthy foods | Chocolate  - Cadbury ‘Bitsa Wispa’ | ~269 | 554 | 34.2 |
|  | Biscuits (mini)  - Fox’s mini malted milk biscuits | ~158 | 484 | 21.4 |
|  | Crisps  - Tesco’s ready salted crisps | ~76 | 550 | 36.3 |
| Healthy foods | Grapes  - green grapes | ~387 | 70 | 0.1 |
|  | Carrot batons  - pre-cut carrots | ~279 | 42 | 0.3 |
|  | Rice cakes (mini)  - Boots’ organic plain rice cakes | ~57 | 388 | 3 |
| Novel unhealthy food | Cheese Bites  - ASDA’s cheese bites | ~172 | 536 | 29.2 |
| Novel healthy food | Breadsticks (mini)  - ASDA’s mini breadsticks | ~110 | 413 | 7.4 |

**Additional Analyses, Study 2**

**Group Differences**

Training groups were well matched for gender ratio, age, BMI, initial RS score, hours since food consumption, food ratings (craving, food liking, food consumption frequencies) and state measures of hunger and mood (all *F*<2.06, all *p*s>0.09, all *ƞ*²s <0.04; a Chi square analysis for gender was not performed due to the small number of males in each group). See Supplementary Table 6 for descriptive statistics and significance tests.

**Supplementary Table 6.** Group characteristics and between-group significance tests for Study 2 (SE within parentheses).

|  | Stop  (*n*=43) | Double-response  (*n*=42) | No-go  (*n*=34) | Go  (*n*=32) | Observe  (*n*=32) | *F*= | *p=* |
| --- | --- | --- | --- | --- | --- | --- | --- |
| Gender (% female) | 93.0 | 90.5 | 91.2 | 90.6 | 90.6 |  |  |
| Age | 20.56 (0.46) | 21.79 (0.94) | 20.59 (0.43) | 23.66 (1.35) | 21.5 (0.89) | 2.05 | 0.09 |
| BMI | 24.17 (0.66)^2^ | 24.89 (0.76)^1^ | 23.49 (0.53)^1^ | 24.92 (0.68) | 23.7 (0.68) | 0.91 | 0.46 |
| RS | 18.64 (0.5)^1^ | 17.83 (0.43) | 18.76 (0.6) | 17.88 (0.48) | 18.63 (0.53) | 0.82 | 0.52 |
| Hours since food | 5.36 (0.54)^4^ | 6.76 (0.88)^4^ | 5.13 (0.57) | 6.3 (0.91) | 5.73 (0.65) | 0.88 | 0.48 |
| Hunger | 4.75 (0.31) | 4.71 (0.34)^1^ | 4.99 (0.33) | 4.98 (0.34)^1^ | 4.88 (0.32) | 0.16 | 0.96 |
| Fullness | 1.71 (0.26) | 1.78 (0.26)^1^ | 1.75 (0.27) | 1.74 (0.33) | 1.56 (0.27) | 0.09 | 0.99 |
| Desire to eat | 5.29 (0.33) | 5.09 (0.33) | 5.55 (0.36) | 5.32 (0.41) | 5.52 (0.43) | 0.26 | 0.90 |
| Positive affect | 23.57 (1.06)^1^ | 25.02 (0.99) | 24.09 (1.15)^1^ | 25.78 (1.13) | 25.75 (1.34) | 0.77 | 0.55 |
| Negative affect | 13.21 (0.48)^1^ | 12.71 (0.44) | 12.52 (0.5)^1^ | 13.09 (0.7) | 13.41 (0.74) | 0.40 | 0.81 |
| Healthy food liking | 6.68 (0.26)^9^ | 6.72 (0.26)^11^ | 6.98 (0.31)^8^ | 6.22 (0.35)^8^ | 6.97 (0.3) | 0.98 | 0.42 |
| Unhealthy food liking | 7.1 (0.26)^9^ | 7.21 (0.26)^12^ | 7.26 (0.28)^8^ | 7.31 (0.29)^8^ | 7.15 (0.26) | 0.10 | 0.98 |
| Healthy food consumption freq. | 3.78 (0.21)^9^ | 3.75 (0.2)^11^ | 3.58 (0.17)^8^ | 3.34 (0.17)^8^ | 3.78 (0.18) | 0.88 | 0.48 |
| Unhealthy food consumption freq. | 3.99 (0.17)^9^ | 3.81 (0.22)^11^ | 3.84 (0.18)^8^ | 3.96 (0.21)^8^ | 3.69 (0.21) | 0.38 | 0.82 |
| FCT | 2.79 (0.17)^7^ | 2.97 (0.15)^4^ | 3.25 (0.15)^2^ | 3.09 (0.12)^3^ | 3.1 (0.15)^1^ | 1.32 | 0.26 |

Note. Superscript denotes the number of participants missing for that variable. RS= Restraint Scale; BMI= body mass index. A Chi square test was not calculated for gender ratio due to the small number of males in each group.

**Training Data Analysis**

On average participants in the stop group successfully inhibited their responses on 61.89% (*SE*=2.20; see Supplementary Table 7) of signal trials (compared to 54.79% in Study 1; *p*=0.004; although the percentage of successful inhibitions for target foods was not significantly different [67.52%] from Study 1 [69.76%], *p*=0.42). Participants in the double-response group responded correctly to signals on 95.27% (*SE*=0.55) of trials. For the no-go group, participants successfully withheld their responses on 94.77% (*SE*=0.63) of trials; this was significantly higher than for the stop group (*t*(48.74) =14.36, *p*<0.001, *d*=3.12). Performance on no-signal trials showed evidence of proactive slowing (Verbruggen & Logan, 2009) in the stop and no-go groups, compared to the double-response and go groups (GoRT: *p*<0.001, *ƞ*²=0.47). Post-hoc follow-up tests also showed a significant slowing in the stop group compared to the no-go group (*p*=0.002; *d*= 0.73). There was also a significant difference between groups for the percentage of incorrect responses on no-signal trials (*p*=0.002, *ƞ*²=0.1). This result appears to be due to a minority of participants in the stop group who missed responses on more than 8% of trials.

**Consumption Data Analysis**

***Stop-Signal vs. Double-Response Training***

Participants in the stop group consumed significantly fewer calories than participants in the double-response group (*p*=0.04, *ƞ*_p_²=0.05; see Figure 4). However, training condition did not significantly interact with food type (*p*=0.27, *ƞ*_p_²=0.02) and the three-way interaction between training condition, food type and food novelty was also non-significant (*p*=0.36, *ƞ*_p_²=0.01). There were also significant main effects of food type (*p*<0.001, *ƞ*_p_²=0.56), and food novelty (*p*=0.001, *ƞ*_p_²=0.13) with participants consuming significantly more calories from unhealthy compared to healthy foods and from old compared to new foods. All results and statistical tests are provided in Supplementary Tables 8 and 9.

A Bayesian analysis on *total* calorie consumption revealed substantial evidence for the experimental hypothesis (H1), that stop training reduces food consumption relative to double-response training (B=3.73; mean difference=93.76, SE of the difference=46.39; Dienes, 2011, 2014; see Figure 4b). This finding reflected an 18.8% difference in total calorie intake between the two groups. A JZS Bayes factor also favoured H1 although evidence was weak (B=1.32).

***Go/No-go vs. Go Training***

Participants in the no-go group consumed significantly fewer calories than those in the go group (*p*<0.001, *ƞ*_p_²= 0.21; see Figure 4). This effect was qualified by a significant interaction between training condition and food type (*p*<0.001, *ƞ*_p_²=0.25); pairwise comparisons revealed that participants in the no-go group consumed significantly fewer unhealthy calories than the go group (*p<*0.001, *ƞ*_p_²=0.24), however, there was no statistically significant difference in the consumption of healthy calories (*p*=0.98, *ƞ*_p_²<0.001). The three-way interaction between condition, food type and food novelty was non-significant (*p*=0.55, *ƞ*_p_² =0.01). The main effect of food type was significant reflecting a greater consumption of calories from unhealthy foods compared to healthy foods (*p*<0.001, *ƞ*_p_² =0.7). All results and statistical tests are provided in Supplementary Tables 8 and 9.

Bayesian analyses for total calorie intake revealed strong evidence for the experimental hypothesis that consumption would be lower in the no-go group compared to the go group (B=22.30, mean difference =180.86, SE of the difference =52.52; Dienes, 2011, 2014; see Figure 4b). This reflected a 32.37% difference in total calorie intake between the two groups. The JZS Bayes factor was also strongly in favour of H1 (B = 30.05).

**Consumption Data Analysis** – *Healthy Food Consumption in Grams*

For the analysis of healthy food consumption, in grams, there was no main effect of condition (*F*(4,178)=1.17, *p=*0.33, *ƞ*_p_²=0.03) and no interaction between condition and food novelty significant (*F*(4,178)=1.55, *p=*0.19, *ƞ*_p_²=0.03).

**Debrief Analysis**

Awareness of the stimulus mappings was assessed for participants in the stop, double-response and no-go groups. Participants were categorised as ‘aware’ (signals mapped onto unhealthy foods), ‘partially aware’ (signals mapped onto food in general) and ‘unaware’. In the stop training group 45% of participants were considered aware, 32.5% were partially aware and 22.5% were not aware. In the double-response group 25% were considered aware, 22.5% partially aware and 52.5% not aware (awareness of food associations in the stop-signal task was greater in Study 2 compared to Study 1 and was more similar to the results of Lawrence et al., 2015a – a result which could be explained by the removal of interblock feedback and the increased rate of successful inhibition). In the no-go group 37.5% of participants were aware and 62.5% were not aware (see Supplementary Table 10 for all debrief frequency data). A Chi square test revealed a statistically significant difference in these frequencies (*χ^2^*(4)=19.33, *p=*0.001, *V*=0.29), reflecting the finding that more participants were aware of the association between unhealthy food and signals in the stop group compared to the double-response and no-go groups. Similarly, fewer participants were classified as not aware in the stop group compared to the double-response and no-go groups. These findings may reflect an increased cognitive load or increased attention in the stop-signal training task whereas performance on the go/no-go task may be more automatic. To see whether awareness had any influence on intake, a 3x2x2x2 mixed ANOVA (between subjects factors: *training condition,* stop, double-response and no-go; *awareness,* aware or not aware; within subjects factors: *food type,* unhealthy or healthy and *food novelty,* old or new) was performed. Due to the small number of participants who were categorised as partially aware the aware groups were collapsed to form one aware group. The results of this analysis revealed no significant main effect of awareness on food consumption (*F*(1,106)=1.22, *p*=0.27, *ƞ*_p_²=0.01) and no statistically significant interactions involving awareness (all *F*s<1.25; all *p*s>0.29; all *ƞ*_p_²s<0.023).

Participants were also probed for awareness of the study’s aims during debrief. Only one participant in the double-response group correctly guessed the aim of the study and was excluded from all analyses. No other participants indicated that they were aware of the study’s aims or that food consumption was being measured. When asked whether they thought that the task had any influence on food intake or performance during the study, the majority of participants (69%) did not report any effects of training and the remaining 31% reported diverse reactions (see Supplementary Table 10).

**Supplementary Table 7.** Training data and statistical comparisons for each condition (SE within parentheses).

|  | Stop  (*n*=43) | Double-response  (*n*=42) | No-go  (*n*=34) | Go  (*n*=32) | *F*= | *p=* | *ƞ*_p_²= |
| --- | --- | --- | --- | --- | --- | --- | --- |
| Correct signal responses (%) | 61.89 (2.2) | 95.27 (0.55) | 94.77 (0.63) |  |  |  |  |
| GoRT | 604.19 (21.48) | 420.82 (10.07) | 522.95 (12.19) | 380.71 (12.57) | 43.43 | <0.001 | 0.47 |
| Stop vs. double-response |  |  |  |  |  | <0.001 |  |
| No-go vs. go |  |  |  |  |  | <0.001 |  |
| Stop vs. no-go |  |  |  |  |  | 0.002 |  |
| Double-response vs. go |  |  |  |  |  | 0.45 |  |
| Incorrect no-signal responses (%) | 2.84 (0.55) | 1.25 (0.15) | 0.92 (0.24) | 1.9 (0.37) | 5.32 | 0.002 | 0.1 |
| Stop vs. double-response |  |  |  |  |  | 0.01 |  |
| No-go vs. go |  |  |  |  |  | 0.53 |  |
| Stop vs. no-go |  |  |  |  |  | 0.003 |  |
| Double-response vs. go |  |  |  |  |  | 1.0 |  |

Note. The observe group is not included as they did not make any responses during the training task.

**Supplementary Table 8.** Food consumption data in kCal for each training condition (SE within parentheses).

|  |  | Stop  (*n*=43) | Double-response  (*n*=42) | No-go  (*n*=34) | Go  (*n*=32) | Observe  (*n*=32) |
| --- | --- | --- | --- | --- | --- | --- |
| Total |  | 404.93 (27.57) | 498.68 (37.51) | 377.79 (35.18) | 558.65 (39.17) | 442.06 (39.1) |
| Means: |  |  |  |  |  |  |
| Unhealthy | Old | 82.91 (8.35) | 105.38 (11.11) | 75.06 (10.42) | 116.19 (11.4) | 88.96 (11) |
|  | New | 59.23 (10.46) | 73.28 (11.3) | 55.18 (10.38) | 121.1 (16.64) | 67.7 (14.8) |
| Healthy | Old | 27.94 (2.38) | 27.92 (2.49) | 28.46 (3.12) | 24.33 (2.27) | 31.26 (3.01) |
|  | New | 13.13 (2.44) | 25.49 (6.21) | 12.06 (3.18) | 16 (2.75) | 13.68 (2.67) |

**Supplementary Table 9.** Results of statistical analyses for food consumption across training conditions.

|  | Stop versus Double-response | | | No-go versus Go | | | All training groups | | |
| --- | --- | --- | --- | --- | --- | --- | --- | --- | --- |
|  | *F*= | *p=* | *ƞ*_p_² | *F*= | *p=* | *ƞ*_p_² | *F*= | *p=* | *ƞ*_p_² |
| Condition | 4.42 | 0.04 | 0.05 | 16.70 | <0.001 | 0.21 | 5.35 | <0.001 | 0.11 |
| Food Type | 107.50 | <0.001 | 0.56 | 151.81 | <0.001 | 0.70 | 271.89 | <0.001 | 0.60 |
| Food Novelty | 12.73 | 0.001 | 0.13 | 2.65 | 0.11 | 0.04 | 17.63 | <0.001 | 0.09 |
| Condition * Food Type | 1.23 | 0.27 | 0.02 | 21.24 | <0.001 | 0.25 | 5.75 | <0.001 | 0.11 |
| Condition * Food Novelty | 0.04 | 0.85 | <0.001 | 1.82 | 0.18 | 0.03 | 0.81 | 0.52 | 0.02 |
| Food Type * Food Novelty | 2.96 | 0.09 | 0.03 | 0.13 | 0.73 | 0.002 | 0.69 | 0.41 | 0.004 |
| Condition * Food Type * Food Novelty | 0.86 | 0.36 | 0.01 | 0.37 | 0.55 | 0.01 | 0.81 | 0.52 | 0.02 |

**Supplementary Table 10.** Results of the debrief analysis. Results show frequencies of responses across training conditions.

|  | Stop  (*n*=43) | Double-response  (*n*=42) | No-go  (*n*=34) | Go  (*n*=32) | Observe  (*n*=32) |
| --- | --- | --- | --- | --- | --- |
| Awareness of stimulus mappings |  |  |  |  |  |
| Aware | 18 (45%) | 10 (25%) | 12 (37.5%) | - | - |
| Partially aware | 13 (32.5%) | 9 (22.5%) | 0 | - | - |
| Not Aware | 9 (22.5%) | 21 52.5%) | 20 (62.5%) | - | - |
| Influence of training |  |  |  |  |  |
| Unhealthy food wanting | 2 | 1 | 1 | 4 | 10 |
| Healthy food wanting | 1 | 0 | 0 | 3 | 3 |
| Hunger, attention to or thinking about food | 2 | 1 | 2 | 1 | 7 |
| Self-control | 4 | 1 | 0 | 0 | 1 |
| Mood | 1 | 2 | 0 | 1 | 3 |
| Performance, alertness | 3 | 3 | 2 | 0 | 1 |

**Additional References**

Attwood, A. S., O’Sullivan, H., Leonards, U., Mackintosh, B., & Munafò, M. R. (2008). Attentional bias training and cue reactivity in cigarette smokers. *Addiction*, *103*, 1875-1882. doi:10.1111/j.1360-0443.2008.02335.x

Best, M., Lawrence, N. S., Logan, G. D., McLaren, I. P. L., & Verbruggen, F. (2015). Should I Stop or Should I Go? The Role of Associations and Expectancies. *Journal of Experimental Psychology: Human Perception and Performance*. http://doi.org/10.1037/xhp0000116

Boot, W. R., Simons, D. J., Stothart, C., & Stutts, C. (2013). The pervasive problem with placebos in psychology: Why active control groups are not sufficient to rule out placebo effects. *Perspectives on Psychological Science*, *8*, 445–454. doi:10.1177/1745691613491271

Faul, F., Erdfelder, E., Lang, A.-G., & Buchner, A. (2007). G*Power 3: A flexible statistical power analysis program for the social, behavioral, and biomedical sciences. *Behavior Research Methods*, *39*, 175–91.

Field, M., Duka, T., Eastwood, B., Child, R., Santarcangelo, M., & Gayton, M. (2007). Experimental manipulation of attentional biases in heavy drinkers: Do the effects generalise? *Psychopharmacology*, *192*, 593–608. DOI 10.1007/s00213-007-0760-9

Field, M., Duka, T., Tyler, E., & Schoenmakers, T. (2009). Attentional bias modification in tobacco smokers. *Nicotine & Tobacco Research*, *11*, 812–22. doi:10.1093/ntr/ntp067

Greenwald, A. G., Nosek, B. A., & Banaji, M. R. (2003). Understanding and using the implicit association test: I. An improved scoring algorithm. *Attitudes and Social Cognition, 85,* 197-216. DOI: 10.1037/0022-3514.85.2.197

Hogarth, L., Dickinson, A., Hutton, S. B., Bamborough, H., & Duka, T. (2006). Contingency knowledge is necessary for learned motivated behaviour in humans: relevance for addictive behaviour. *Addiction*, *101*, 1153–66. doi:10.1111/j.1360-0443.2006.01459.x

Houben, K., Roefs, A., & Jansen, A. (2012c). Guilty pleasures II : Restrained eaters’ implicit preferences for high, moderate and low-caloric food. *Eating Behaviors*, *13*, 275–277. doi:10.1016/j.eatbeh.2012.03.007

Kakoschke, N., Kemps, E., & Tiggemann, M. (2014). Attentional bias modification encourages healthy eating. *Eating Behaviors*, *15*, 120–124. doi:10.1016/j.eatbeh.2013.11.001

Logan, G. D., Schachar, R. J., & Tannock, R. (1997). Impulsivity and inhibitory control. *Psychological Science, 8*, 60-64.

Robinson, E., Kersbergen, I., Brunstrom, J. M., & Field, M. (2014). I’m watching you: awareness that food consumption is being monitored is a demand characteristic in eating-behaviour experiments. *Appetite*, *83*, 19–25. doi:10.1016/j.appet.2014.07.029

Tabachnick, B. G., & Fidell, L. S. (2007). Using multivariate statistics (5th ed.). Boston: Allyn and Bacon.

Verbruggen, F., & Logan, G. D. (2009). Proactive adjustments of response strategies in the stop-signal paradigm. *Journal of Experimental Psychology. Human Perception and Performance*, *35*, 835–54. doi:10.1037/a0012726

1. Effect size *f* = $\frac{\sigma means}{\sigma}$; suggested values for small, medium and large effects are 0.1, 0.25 and 0.4, respectively (Cohen, 1988).

   *f*$= \sqrt{\frac{ƞ_{p}^{2}}{(1-ƞ_{p}^{2})}}$ [↑](#footnote-ref-1)
2. http://www.lifesci.sussex.ac.uk/home/Zoltan_Dienes/inference/bayes_normalposterior.swf [↑](#footnote-ref-2)
3. http://www.lifesci.sussex.ac.uk/home/Zoltan_Dienes/inference/bayes_factor.swf [↑](#footnote-ref-3)
4. http://pcl.missouri.edu/ [↑](#footnote-ref-4)
